# Treatment of colonic varices with a superior mesenteric venous stent: a case report describing a unique approach

**DOI:** 10.1093/gastro/goab003

**Published:** 2021-02-04

**Authors:** Sandra Naffouj, Mustafa Al-Shammari, Reena Salgia

**Affiliations:** 1 Department of Internal Medicine, Henry Ford Hospital, Detroit, MI, USA; 2 Division of Gastroenterology and Hepatology, Department of Medicine, Henry Ford Hospital, Detroit, MI, USA

## Introduction 

Ectopic varices are a rare cause of gastrointestinal (GI) bleeding that may occur in the GI tract outside the esophageal and gastric distribution [1]. The majority of these cases are rectal varices in cirrhotic patients with portal hypertension. Colonic varices (CV) are less common than rectal, duodenal, and small-bowel varices [2]. There are no clear guidelines for managing CV given the rarity of this finding [3]. We report a case of massive lower-GI bleeding due to CV in a patient with non-alcoholic steatohepatitis (NASH) without cirrhosis or portal hypertension secondary to porto-mesenteric venous thrombosis that was successfully treated with superior mesenteric venous (SMV) stenting. Given the extent of the mesenteric thrombosis, treatment modalities like transjugular intrahepatic portosystemic shunt (TIPS) and balloon-occluded retrograde transvenous obliteration (BRTO) were not possible.

## Case report

**Figure 1.**
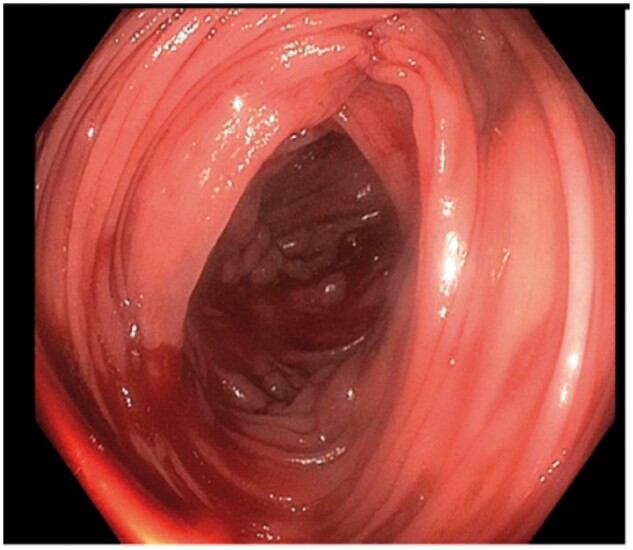
Endoscopic view of bleeding colonic varices.

A 57-year-old man with NASH without cirrhosis presented to a satellite hospital with acute onset of painless hematochezia for 1 day. He had unprovoked deep venous thrombosis with a negative hypercoagulable workup and portal-vein thrombosis on Apixaban diagnosed 18 months prior to presentation. He was tachycardic and mildly hypotensive. There was no evidence of chronic liver disease on exam. The hemoglobin level was 130 g/L and subsequently decreased to 80 g/L, while the platelet count was 160 × 10^9^/L. Laboratory tests revealed the following values: INR, 1.13; lactate, 1 mmol/L; albumin, 3.7 g/dl; and liver enzymes were within normal limits. Anticoagulation was discontinued and he was resuscitated using intravenous (IV) crystalloids. Esophagogastroduodenoscopy (EGD) revealed small non-bleeding esophageal varices. Colonoscopy revealed a complex ascending colonic varix with active bleeding (Figure 1).

He was treated with IV octreotide at the satellite hospital at which he presented and then transferred to our tertiary institution for escalation of care. Liver-protocol computed tomography (CT) was performed for TIPS evaluation and revealed a steatotic liver and collateralized chronically thrombosed SMV with patent portal vein. He was deemed not to be a candidate for either TIPS or BRTO in the setting of chronic SMV thrombosis. Colonic resection was not recommended, since it was unlikely to prevent future bleeding with significant collaterals.

He continued to have hematochezia with Hgb nadir of 78 g/L. A trans-hepatic portogram performed by interventional radiology revealed an elevated absolute portal pressure of 18 mmHg. Digital subtraction images confirmed the site of the SMV occlusion at the confluence of two large SMV branches, in addition to retrograde flow through the large right CV. The occluded SMV was cannulated through a left patent branch using a 4-French angled glide catheter and a stiff Glidewire. A Cobra 2 catheter and a stiff Glidewire were used to cannulate the right SMV branch at the confluence. Recanalization of the occluded SMV (Supplementary Figure 1) was achieved by using 5  × 40 mm balloon angioplasty to 5 mm, then two overlapping 10 × 40 mm self-expanding stents were successfully deployed into the origin of the right SMV branch, extending proximally into the portal splenic confluence. The stents were then post-dilated using balloon angioplasty to 8 and 10 mm. Follow-up venography showed antegrade flow in the right SMV branch into the SMV stent with a marked reduction in filling of the right CV (Supplementary Figure 2). The procedure was terminated after restoration of the SMV flow and the portal pressure was not measured post-procedurally. Liver biopsy showed steatohepatitis with stage 2/4 fibrosis. Apixaban and low-dose aspirin were started. Two months after discharge, the patient had no recurrent bleeding and CT showed patent SMV stents (Supplementary Figure 3). A plan for surveillance was determined using cross-sectional imaging every 6 months for 2 years.

## Discussion

CV are dilated portosystemic venous collaterals in the colonic submucosa that comprise a rare cause of GI bleeding. CV have a reported incidence rate of 0.07% in the general population [4]. They are usually located in the distribution of the inferior mesenteric vein (IMV) (66%), SMV distribution (26%), and mixed supply areas [5]. CV are most commonly diagnosed in cirrhotic patients with portal hypertension [6]. Non-cirrhotic etiologies include congestive heart failure, portomesenteric venous compromise, splenic venous thrombosis, inherited vascular anomalies, or they can be idiopathic [7].

Patients with CV usually present with intermittent hematochezia or massive rectal bleeding. The gold standard for diagnosis is selective mesenteric angiography, which can be therapeutic and diagnostic [4]. However, colonoscopy is more commonly performed as a first step for lower-GI-bleeding evaluation. Detection via colonoscopy can be obscured by polyps, malignant masses, active bleeding, or may be missed if the varices are flattened with insufflation [8].

Endoscopic therapies, TIPS, BRTO, angiographic embolization, and even colonic resection have been described in CV management [9]. However, due to limited data, standard management is still not well defined. The treatment typically focuses on symptom control and management of the underlying medical conditions causing the varices. Endoscopic management includes injections of cyanoacrylate, sclerotherapy, and argon plasma coagulation to control active bleeding. In the setting of portal hypertension, TIPS and angiographic embolization are more effective and result in normalization of the portal-system pressure, which can prevent future bleeding. Zhang *et al*. [10] reported higher rates of success with TIPS than that with endoscopic intervention in cirrhotic patients. However, TIPS was associated with worsening hepatic encephalopathy.

The present case was unique in presentation and management, and, to our knowledge, there has been no reported case of CV treatment with a similar approach. TIPS was not performed, as it would not have relieved the pressure of the CV, and BRTO would have likely increased the risk of mesenteric ischemia. Anticoagulation alone was not feasible given the ongoing bleeding and colonic resection carried a high risk of mortality and of recurrent bleeding from collaterals. We had an excellent outcome with the least invasive approach possible.

## Conclusions

CV may present with lower-GI bleeding, ranging from mild to life threatening, which makes early diagnosis of extreme importance. Management differs based on the available medical expertise, the underlying etiology of the varices, and the patient’s clinical presentation. Further studies are needed to establish a clear stepwise approach for management of these rare but dangerous findings. In cases of CV with severe mesenteric thrombosis and portal hypertension, treatment with an SMV stent may be considered.

## Supplementary Data

Supplementary data is available at *Gastroenterology Report* online.

## Authors’ contributions

S.N. and M.A. conceived of the case, collected data, and drafted the manuscript. R.S. supervised the findings of this work and provided critical revision for the manuscript. All authors read and approved the final manuscript.

## Funding

There are no financial disclosures to report.

## Supplementary Material

goab003_Supplementary_Data
