# Supplementary figures and images for: Treatment of colonic varices with a superior mesenteric venous stent: a case report describing a unique approach
Source: Gastroenterol Rep (Oxf). 2021 Feb 4;9(6):597–600. doi: 10.1093/gastro/goab003 (PMC8677559; doi:10.1093/gastro/goab003)

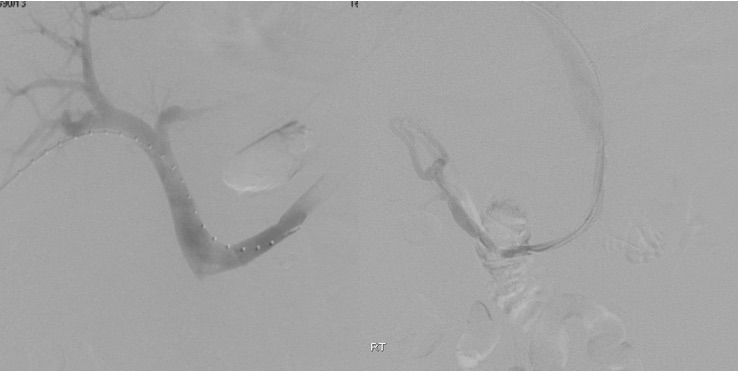

Supplement: goab003_Supplementary_Data [file goab003_supplementary_data.zip › 2020-221 Supplementary Figure_1.jpg]

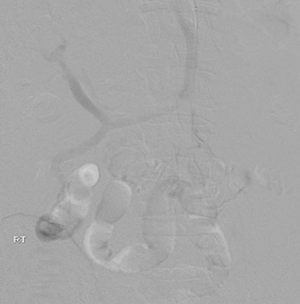

Supplement: goab003_Supplementary_Data [file goab003_supplementary_data.zip › 2020-221 Supplementary Figure_2.png]

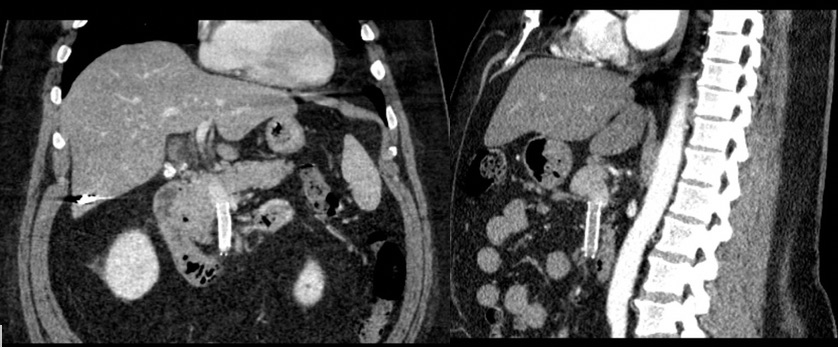

Supplement: goab003_Supplementary_Data [file goab003_supplementary_data.zip › 2020-221 Supplementary Figure_3.jpg]
